# Quantitative analysis of intracranial electrocorticography signals using the concept of statistical parametric mapping

**DOI:** 10.1038/s41598-019-53749-3

**Published:** 2019-11-22

**Authors:** Hirotaka Motoi, Jeong-Won Jeong, Csaba Juhász, Makoto Miyakoshi, Yasuo Nakai, Ayaka Sugiura, Aimee F. Luat, Sandeep Sood, Eishi Asano

**Affiliations:** 1 Department of Pediatrics, Children’s Hospital of Michigan, Wayne State University, Detroit Medical Center, Detroit, MI 48201 USA; 2 Department of Neurology, Children’s Hospital of Michigan, Wayne State University, Detroit Medical Center, Detroit, MI 48201 USA; 3 Department of Neurosurgery, Children’s Hospital of Michigan, Wayne State University, Detroit Medical Center, Detroit, MI 48201 USA; 4 Swartz Center for Computational Neuroscience, Institute for Neural Computation, University of California San Diego, La Jolla, CA 92093 USA; 5 Department of Pediatrics, Yokohama City University Medical Center, Yokohama, 2320024 Japan

**Keywords:** Epilepsy, Epilepsy

## Abstract

Statistical parametric mapping (SPM) is a technique with which one can delineate brain activity statistically deviated from the normative mean, and has been commonly employed in noninvasive neuroimaging and EEG studies. Using the concept of SPM, we developed a novel technique for quantification of the statistical deviation of an intracranial electrocorticography (ECoG) measure from the nonepileptic mean. We validated this technique using data previously collected from 123 patients with drug-resistant epilepsy who underwent resective epilepsy surgery. We determined how the measurement of statistical deviation of modulation index (MI) from the non-epileptic mean (rated by z-score) improved the performance of seizure outcome classification model solely based on conventional clinical, seizure onset zone (SOZ), and neuroimaging variables. Here, MI is a summary measure quantifying the strength of *in-situ* coupling between high-frequency activity at >150 Hz and slow wave at 3–4 Hz. We initially generated a normative MI atlas showing the mean and standard deviation of slow-wave sleep MI of neighboring non-epileptic channels of 47 patients, whose ECoG sampling involved all four lobes. We then calculated ‘MI z-score’ at each electrode site. SOZ had a greater ‘MI z-score’ compared to non-SOZ in the remaining 76 patients. Subsequent multivariate logistic regression analysis and receiver operating characteristic analysis to the combined data of all patients revealed that the full regression model incorporating all predictor variables, including SOZ and ‘MI z-score’, best classified the seizure outcome with sensitivity/specificity of 0.86/0.76. The model excluding ‘MI z-score’ worsened its sensitivity/specificity to 0.86/0.48. Furthermore, the leave-one-out analysis successfully cross-validated the full regression model. Measurement of statistical deviation of MI from the non-epileptic mean on invasive recording is technically feasible. Our analytical technique can be used to evaluate the utility of ECoG biomarkers in epilepsy presurgical evaluation.

## Introduction

Interictal *spike-and-wave* discharges are classic electrographic biomarkers for diagnosis of epilepsy. Cortical regions showing spike-and-wave discharges on electrocorticography (ECoG) are also known as the irritative zone, which often, but not always, overlaps with the seizure onset zone (SOZ) responsible for habitual seizures in patients with drug-resistant focal epilepsy^1,2^. Interictal spike-and-wave discharges are accompanied by high-frequency activity at >80 Hz (HFA > 80 Hz) to a variable degree^3–6^. HFA > 80 Hz components accompanied by interictal spike-and-wave discharges were suggested to be predictive of SOZ^7^. HFA is herein defined as ***a spectrum*** encompassing both paroxysmal events of ≥six cycles of discrete/organized oscillations visible without a high-pass filter (often referred to as high-frequency oscillations [HFOs]) and instantaneous power increase at high-frequency range containing no oscillations (i.e.: a very sharply-contoured transient)^8–10^. In this clinical study, we did not differentiate these entities of the HFA spectrum, since previous studies did not necessarily prove that the clinical utility of the former type of HFA > 80 Hz is greater than that of the latter, but rather suggested that both types of HFA > 80 Hz are frequently generated by the SOZ^10,11^. Incomplete resection of regions showing high occurrence rate of HFA > 80 Hz was suggested to accurately predict poor postoperative seizure outcome in some patient cohorts but not in others^12–18^. Suboptimal outcome prediction by HFA rate measures was partly attributed to the notion that HFA is also generated by ***non-epileptic recording sites***, defined as those not involved in the SOZ, interictal spike discharges, or epileptogenic lesions^19,20^. Thus, some investigators have proposed the need for statistical assessment of HFA relative to anatomically accurate normative standards for better localization of the epileptogenic zone^21^.

Based on the observations that interictal spike-and-wave discharges are accompanied by HFA > 150 Hz coupled with local slow wave in a stereotypical manner, we suggest that the ***modulation index (MI)***, quantifying the phase-amplitude coupling between interictal HFA > 150 Hz and phase of slow wave at 3–4 Hz, would be an excellent surrogate marker of the irritative zone^22,23^. In two independent patient cohorts, MI was correlated with the occurrence rate of HFA, and SOZ was associated with higher MI compared to non-SOZ^22,23^. Thereby, phase-amplitude coupling between HFA and slow wave at 3–4 Hz better distinguished SOZ and non-SOZ compared to those between HFA and slow waves of other frequency bands. Greater MI in the non-removed regions was associated with poor postoperative seizure outcome^24^. The MI algorithm computing the phase-amplitude coupling was initially utilized to determine the physiological ECoG changes related to sensorimotor and cognitive tasks^25^. Thus, one would wonder if MI during resting state might differ across different anatomical structures. Indeed, we previously found that MI during slow-wave sleep in non-epileptic recording sites exhibited an anatomical variability^24^; specifically, occipital sites had higher MI compared to other cortical regions, whereas superior-temporal and superior-frontal sites showed somewhat lower MI. Thus, we believe that the statistical deviation of resting state MI also needs to be assessed to optimize the clinical utility of this ECoG biomarker in epilepsy presurgical evaluation.

***Statistical parametric mapping (SPM)*** has been employed in noninvasive neuroimaging and EEG studies, to readily quantify the deviation of brain activity from the mean among a control population for objective localization of the pathological brain regions associated with a neurological disease of interest^26–28^. The conventional SPM analysis requires data from a control population to calculate the normative mean and standard deviation, but invasive ECoG studies would inevitably lack such a distinct control population. Thus, we developed a novel technique to quantify the statistical deviation of ECoG measure from the mean among neighboring non-epileptic recording sites of a patient cohort, in whom ECoG sampling involved all four lobes^19,20^. We then determined whether the statistical deviation of MI from the non-epileptic mean (rated by z-score of MI [‘MI z-score’]) would accurately classify the SOZ responsible for the generation of habitual seizures in a different patient cohort. We also determined how the addition of ‘MI z-score’ to a multivariate logistic regression model would improve the accuracy of classification of patients achieving surgical success defined as ILAE Class 1 outcome^29^. Finally, we cross-validated the performance of our multivariate logistic regression model using a leave-one-out method^30^.

The novelty of this study includes the generation of an anatomically-accurate normative standard of MI for epilepsy presurgical evaluation. We expected that measurement of statistical deviation of MI from the non-epileptic mean (i.e., normative mean) would be technically feasible, partly because MI is a continuous variable whereas the occurrence rate of HFA or interictal epileptiform activity is a discrete one.

## Methods

### Patients

We studied the patient cohort, identical to that reported in our previous study^24^, consisting of a consecutive series of 123 patients who satisfied the following criteria (mean age: 13 years; range: 4 to 44 years). Thereby, 107 patients were younger than 18 years old. Included patients were those who underwent resective surgery following extraoperative ECoG recording with a sampling rate of 1000 Hz at our institution between January 2007 and October 2016. We excluded patients if (a) the epileptogenic zone was determined to be present independently in both hemispheres based on the non-invasive evaluation, (b) they needed hemispherectomy or hemispherotomy, (c) extensive brain malformations distorting major anatomical landmarks (such as megalencephaly) prevented analysis on the FreeSurfer average brain^31^, (d) postoperative follow-up was shorter than 12 months, (e) prior resective epilepsy surgery was done, or (f) age was <4 years (due to a risk of surface registration errors^32^. The study protocol was approved by the Institutional Review Board at Wayne State University and written informed consent was obtained from patients or guardians of pediatric patients. All experiments were performed in accordance with relevant guidelines and regulations.

### ECoG recording and visualization

ECoG data acquisition methods have been identical to those previously described^24^. Seizure semiology, scalp EEG, and neuroimaging data guided the placement of subdural disk electrodes on the epileptic hemisphere. We placed surface electromyography (EMG) and electrooculography (EOG) electrodes to determine the onset of clinical symptoms and sleep staging^19,22,33^. We recorded ECoG signals at the bedside with a band-pass of 0.016 to 300 Hz for 3 to 7 days and analyzed signals on a common average reference^34,35^. We discontinued antiepileptic drugs (AEDs) and resumed them once SOZ was determined. We clinically defined the SOZ as regions initially exhibiting sustained rhythmic waveforms prior to the onset of habitual seizure symptoms, not explained by sleep state changes, and clearly distinguished from interictal activity^36^. We excluded recording sites affected by artifacts from further analysis. Thus, the subsequent analyses were employed on 12964 electrodes (mean: 105 electrodes per patient).

At an individual level, we created a three-dimensional MRI surface image with the location of each subdural electrode co-registered on it^37^. Each electrode site was spatially normalized using FreeSurfer scripts (http://surfer.nmr.mgh.harvard.edu), and assigned a vertex point spatially compatible to the FreeSurfer average brain coordinate^31,32^. This procedure allowed us to visualize ECoG measures on either individual or FreeSurfer average brain image.

### Surgical decision and measurement of the size of resection

We determined the extent of cortical resection according to the clinical factors, semiology, visual assessment of extraoperative ECoG, extent of lesion, and eloquent areas^36^. We intended to remove regions classified as SOZ, those showing frequent interictal spike-and-wave discharges, and lesions proximal to SOZ when present. If ECoG recording failed to capture ictal events, we planned to remove regions showing frequent interictal spike-and-wave discharges and the associated lesion. We determined the exact resection margin on a case-by-case basis when eloquent cortex overlapped with the regions presumed to be epileptogenic. This is a retrospective observational study, and measurement of MI did not affect our surgical planning.

Following cortical resection and prior to dural closure, we obtained intraoperative photographs to confirm the extent of resection and determined whether all recording sites classified as SOZ were completely removed^24^. Thereby, resection of SOZ sites was considered to be incomplete, if a SOZ site was present on the un-removed gyrus. SOZ sites were considered to be removed entirely if they were localized at the top of a sulcus and the resection was completed up to that sulcus. Using FreeSurfer scripts, the first author, while being blinded to the postoperative seizure outcome, quantified how much percentage of the affected hemisphere was removed (mean of resection size: 13.8%; range of resection size: 0.6 to 91.6%).

### Computation of modulation index (MI) and ‘MI z-score’ based on the normative atlas

EEGLAB Toolbox winPACTv.2.0 (https://sccn.ucsd.edu/eeglab/index.php)^38^ computed MI, the strength of coupling between the HFA > 150 Hz amplitude and the instantaneous phase of local slow wave 3–4 Hz, during 10 earliest available, 30-second epochs of slow-wave sleep. We then assigned MI value averaged across ten 30-second epochs at each recording site.

**Figure 1:**
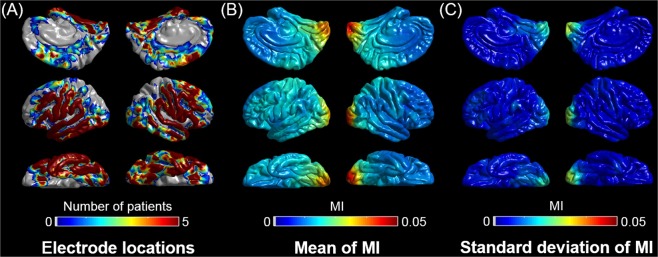
Normative standard of modulation index (MI) during interictal slow-wave sleep. (**A**) The locations of spatially-normalized non-epileptic electrode sites of 47 patients used to generate this normative atlas showing the topography of phase-amplitude coupling between the HFA > 150 Hz amplitude and the phase of local slow wave 3–4 Hz. (**B**) Mean across 30 closest sites. (**C**) Standard deviation across 30 closest sites.

**Figure 2:**
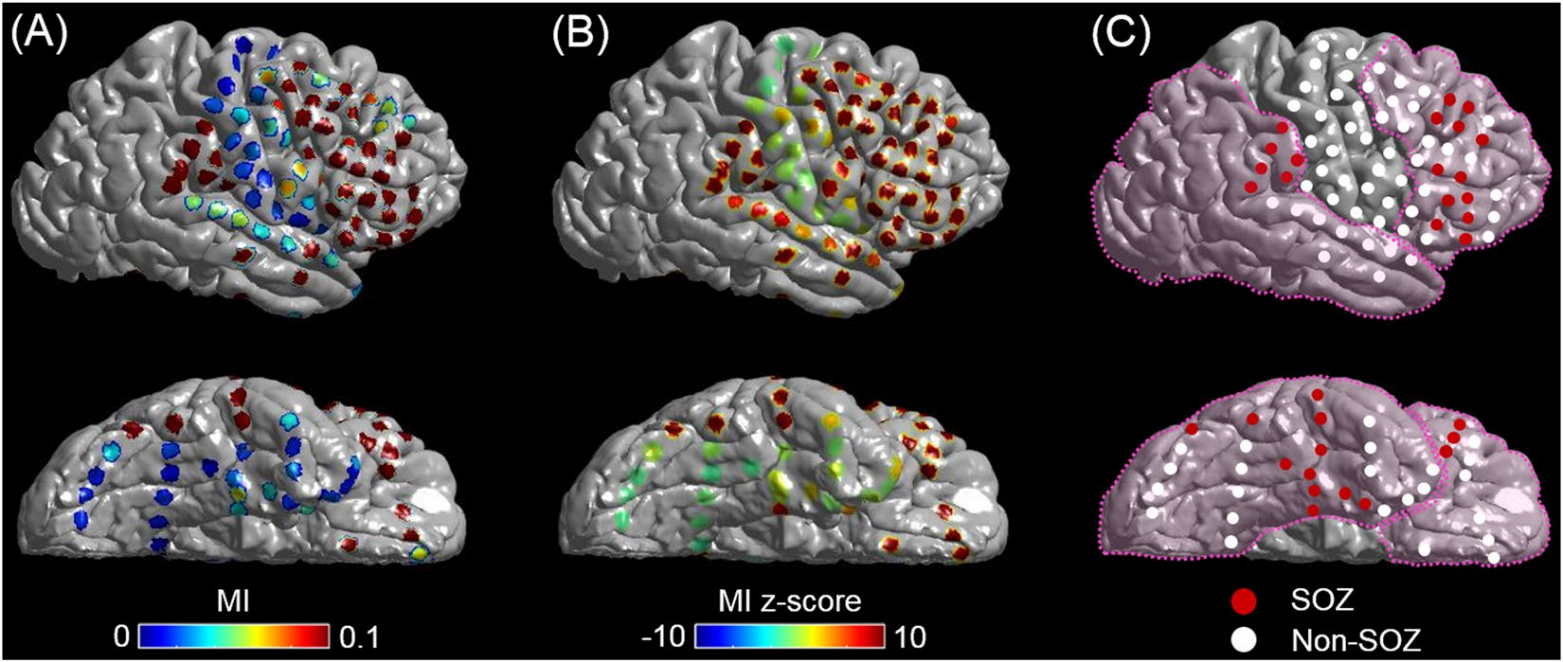
Modulation index (MI) in a 19-year-old girl with drug-resistant focal epilepsy. (**A**) Topography of MI. (**B**) Topography of ‘MI z-score’. (**C**) Location of seizure onset zone (SOZ) is denoted as red electrodes. Dotted line: resection margin. Following subtotal hemispherectomy^23,40^, she achieved ILAE class 1 outcome (follow-up period: 3.4 years).

**Figure 3:**
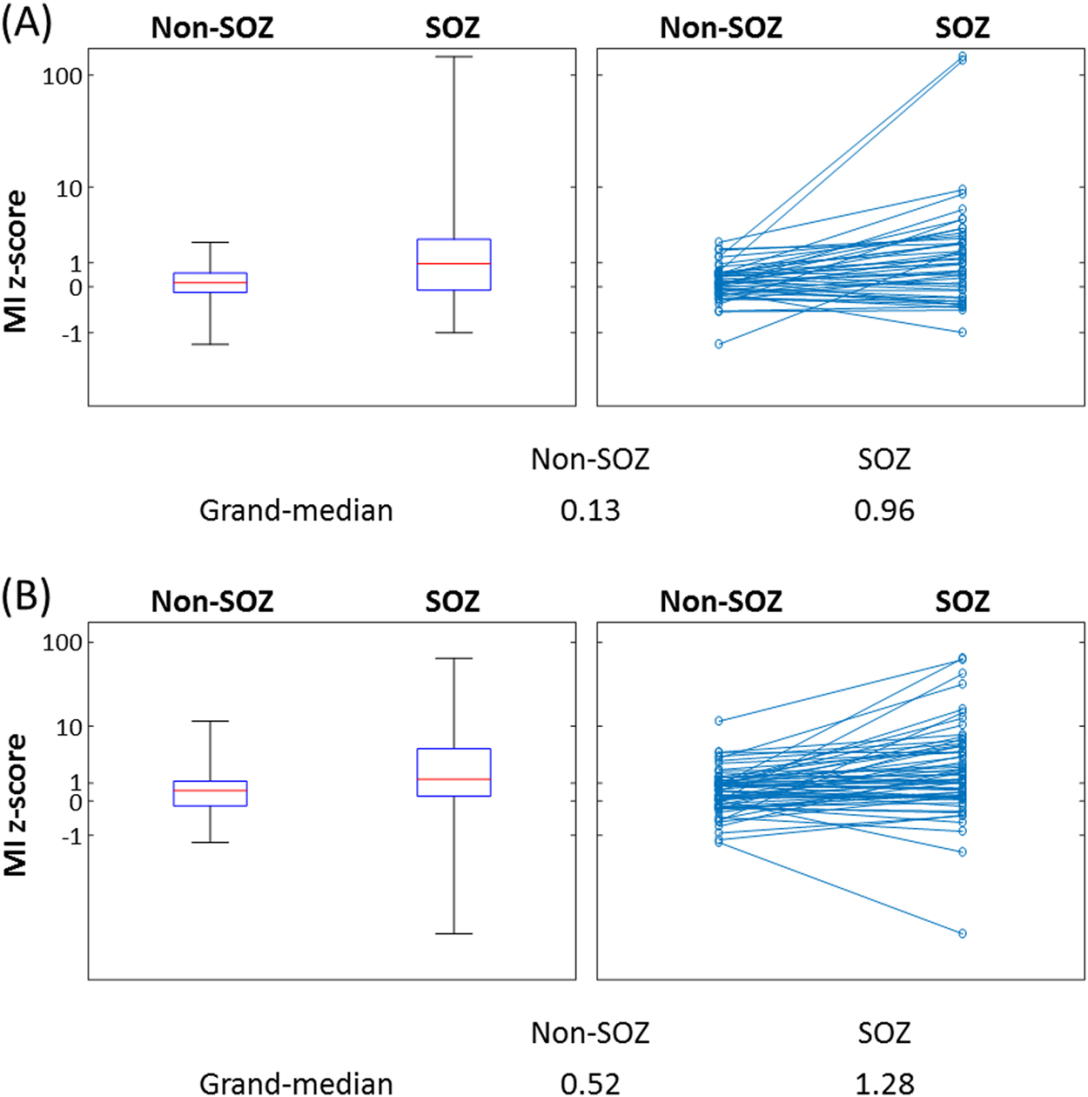
‘MI z-score’ at SOZ and non-SOZ electrodes. (**A**) Data derived from 47 patients, in whom 440 and 4556 electrodes were classified as SOZ and non-SOZ sites, respectively. The box plots denote the median, the 25th and 75th percentiles, and the range of MI z-scores at SOZ and non-SOZ of the 47 patients. Each patient’s median values of ‘MI z-score’ within SOZ and non-SOZ are denoted by dots connected by a line. (**B**) Data derived from 64 patients (out of the 76 patients) who revealed SOZ during extraoperative ECoG recording. Thereby, 790 and 4129 electrodes were classified as SOZ and non-SOZ sites. The box plots are likewise presented.

We generated the anatomically-accurate normative MI atlas showing the topography of phase-amplitude coupling between HFA > 150 Hz and slow wave 3–4 Hz on the FreeSurfer average surface image. For this purpose, we used MI of spatially-normalized non-epileptic sites of 47 patients, in whom electrode placement involved all four lobes and effectively allowed us to complete sleep staging^24^. Thereby, non-epileptic sites were defined as those not involved in the SOZ, interictal spike discharges, or epileptogenic lesions^19,20^. Our MATLAB-based in-house software calculated the mean and standard deviation of MI at each surface model mesh vertex^39^ using MI values at 30 closest spatially normalized non-epileptic sites (Fig. 1). This procedure allowed us to compute ‘MI z-score’, reflecting how much MI was deviated from the non-epileptic mean, at given recording sites of given patients at a given individual brain surface image (Fig. 2). In each patient, we calculated the median values of ‘MI z-score’ within SOZ and non-SOZ. The Wilcoxon signed rank test finally determined if the SOZ was associated with a greater ‘MI z-score’ compared to non-SOZ in the remaining 76 patients (Fig. 3).

### Classification of postoperative seizure outcome using multivariate logistic regression models

**Figure 4:**
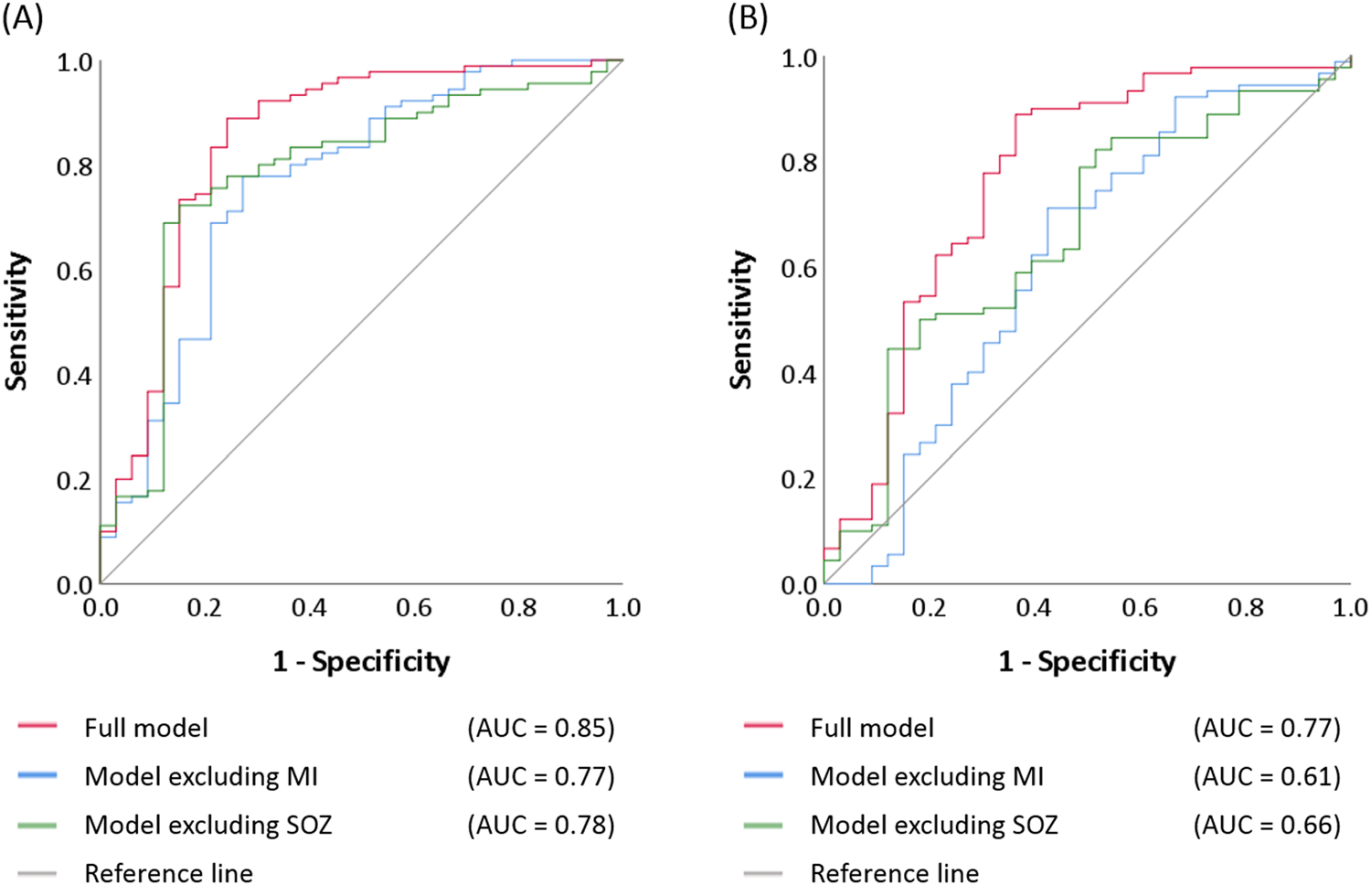
Receiver operating characteristic (ROC) plots. (**A**) ROC plots indicate the model performance to classify surgical success, defined as achievement of ILAE Class-1 outcome within all 123 patients. Red line: Full model incorporating ‘subtraction MI z-score’ in addition to clinical, seizure onset zone (SOZ), neuroimaging variables (Table 1). Blue line: Model excluding MI, which incorporated all predictor variables included in the full model except ‘subtraction MI z-score’. Green line: Model excluding SOZ, which incorporated all predictor variables included in the full model except SOZ variables. Area under the curve (AUC) of 0.5 indicates random classification, whereas 1.0 indicates perfect classification. (**B**) ROC plots with a leave-one-out approach employed.

Using the combined data of all 123 patients, we generated multivariate logistic regression models. This analysis was done with SPSS Statistics 25 (IBM Corp., Chicago, IL, USA), and the significance was set at p-value at 0.05. The goal was to compare the performance of classification of patients achieving surgical success (i.e., ILAE Class 1 outcome) between the full model and that excluding the MI variable. ***The full model*** specifically incorporated ‘age’, ‘gender’, ‘presence of daily seizures’, ‘number of oral AEDs taken immediately prior to extraoperative ECoG recording, ‘affected hemisphere’ (left or right), ‘presence of cortical lesion on MRI’, ‘occurrence of habitual clinical seizure events during extraoperative ECoG recording for localizing SOZ’, ‘incomplete resection of SOZ’, ‘necessity of extra-temporal lobe resection’, ‘size of resection’, and ‘subtraction MI z-score’. It should be emphasized that all of the aforementioned predictor variables can be obtained prior to the completion of the resective surgery. ***‘Subtraction MI z-score’*** was defined as subtraction of ‘MI z-score’ averaged across all preserved sites from ‘MI z-score’ averaged across all resected sites. A given patient would be assigned a large ‘subtraction MI z-score’ if cortical regions with large ‘MI z-score’ were removed and those with small ‘MI z-score’ were preserved. ***The model excluding MI*** incorporated all predictor variables except ‘subtraction MI z-score’. We subsequently employed receiver operating characteristic (ROC) analysis to a model-based probability of surgical success in each patient based on each regression model^36,41,42^. This analysis yielded the accuracy of outcome classification, as rated by the area under the curve (AUC) of a given ROC plot, as well as sensitivity/specificity of outcome classification by each regression model (Fig. 4A).

As a secondary analysis, we explored how critically the SOZ variables contributed to accurate classification of postoperative seizure outcome. Namely, we assessed the accuracy of outcome classification of ***the model excluding SOZ***, which incorporated all predictor variables including ‘subtraction MI z-score’, but excluded SOZ variables (Table 1).

**Table 1:** Multivariate logistic regression models.

Predictor variable	Full model	Odds ratio (95%CI); p-value	Model excluding SOZ
		Model excluding MI	
Age (years)	1.04 (0.96 to 1.13); p = 0.30	1.02 (0.95 to 1.10); p = 0.59	1.05 (0.98 to 1.13); p = 0.20
Gender (1 if male; 0 female)	1.36 (0.49 to 3.78); p = 0.56	1.19 (0.47 to 3.01); p = 0.71	1.44 (0.59 to 3.52); p = 0.42
Daily seizures (1 if present; 0 otherwise)	1.39 (0.41 to 4.74); p = 0.60	1.45 (0.50 to 4.25); p = 0.49	1.57 (0.56 to 4.39); p = 0.39
Number of AEDs	0.39 (0.20 to 0.78); p = 0.008	0.47 (0.25 to 0.86); p = 0.01	0.49 (0.27 to 0.88); p = 0.02
Affected hemisphere (1 if left; 0 if right)	0.69 (0.25 to 1.95); p = 0.49	0.86 (0.34 to 2.18); p = 0.75	0.75 (0.29 to 1.90); p = 0.54
MRI lesion (1 if present; 0 otherwise)	1.20 (0.41 to 3.44); p = 0.74	0.97 (0.37 to 2.52); p = 0.94	1.36 (0.55 to 3.38); p = 0.51
Habitual clinical seizures during ECoG (1 if present; 0 otherwise)	3.93 (0.77 to 19.96); p = 0.10	1.16 (0.24 to 5.52); p = 0.85	not incorporated
Incomplete SOZ resection (1 if incomplete; 0 otherwise)	0.03 (0.005 to 0.17); p = 0.0001	0.10 (0.03 to 0.36); P = 0.0005	not incorporated
Extra-temporal lobe resection (1 if involved; 0 otherwise)	1.33 (0.43 to 4.14); p = 0.62	1.35 (0.46 to 3.96); p = 0.58	0.85 (0.31 to 2.34); p = 0.76
Size of resection (%)	0.98 (0.95 to 1.01); p = 0.25	0.99 (0.97 to 1.01); p = 0.44	0.97 (0.95 to 1.00); p = 0.04
Subtraction MI z-score	1.45 (1.08 to 1.94); p = 0.01	not incorporated	1.23 (1.00 to 1.53); p = 0.05

AEDs: antiepileptic drugs. ECoG: electrocorticography. SOZ: seizure onset zone. MI: modulation index. ‘Subtraction MI z-score’ is defined as subtraction of MI z-score averaged across all preserved sites from MI z-score averaged across all resected sites. 95%CI: 95% confidence interval.

### Effects of different definitions of the non-epileptic mean on the outcome classification

We determined how much the accuracy of the outcome classification, rated by AUC, differed among the full models utilizing the non-epileptic mean based on 10, 20, 30, 40, 50, and 60 closest electrode sites.

### Cross validation of the multivariate logistic regression model

We cross-validated the full model using a leave-one-out approach^30,43^. We estimated the probability of surgical success of each new patient based on the full multivariate logistic regression model incorporating all variables derived from the remaining 122 patients. The AUC of ROC curves determined how much the full model’s performance of outcome classification was altered by the employment of a leave-one-out approach (Fig. 4B).

### Visualization of the performance of outcome classification models at an individual patient level

**Figure 5:**
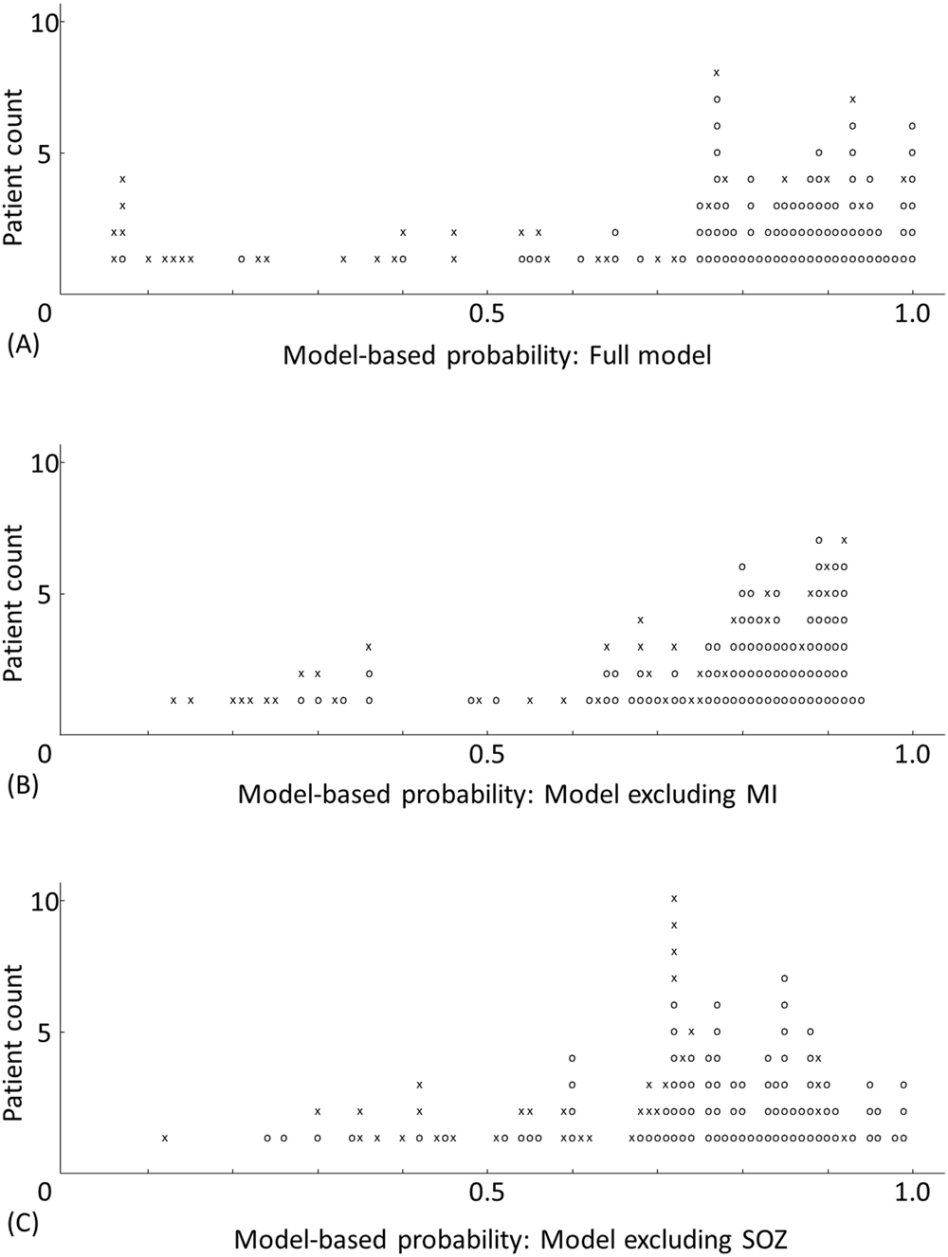
Agreement between the model-based probability and the observed frequency of surgical success. Performance of outcome classification based on (**A**) the full model, (**B**) the model excluding MI, and (**C**) the model excluding SOZ. O (circle): surgical success. × (cross): surgical failure.

**Figure 6:**
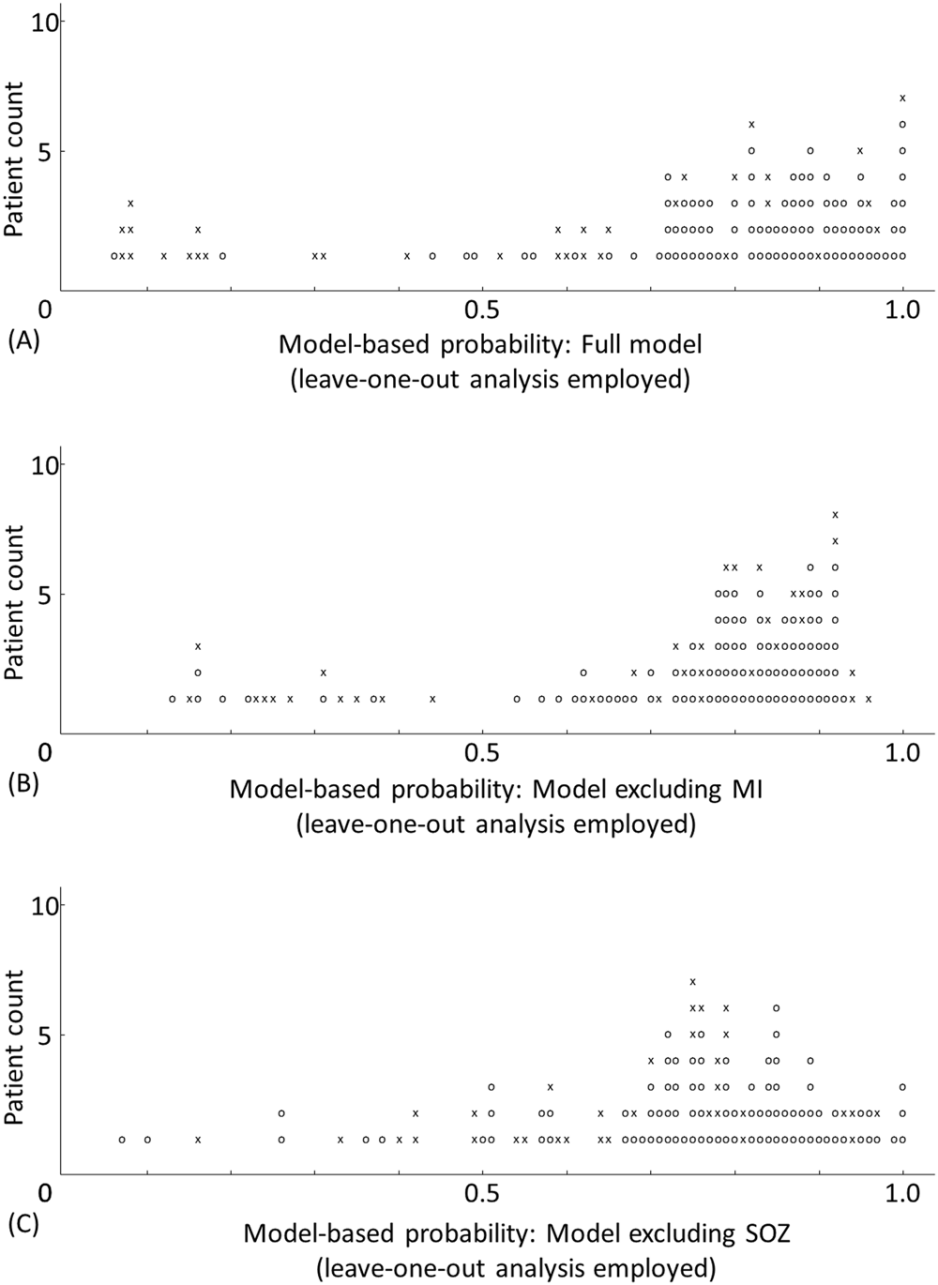
Agreement between the model-based probability and the observed frequency of surgical success with the leave-one-out analysis employed. Performance of outcome prediction based on the leave-one-out multivariate logistic models: (**A**) the full model, (**B**) the model excluding MI, and (**C**) the model excluding SOZ. O (circle): surgical success. × (cross): surgical failure.

We visualized the agreement between the regression model-based probability and the observed frequency of surgical success at an individual patient level (Figs. 5 and 6) to better conceptualize the accuracy of a given regression model in outcome classification.

## Results

A total of 2477 recording sites were classified as non-epileptic and allowed us to generate a normative MI atlas (Fig. 1). SOZ had a greater ‘MI z-score’ compared to non-SOZ in 47 patients whose non-epileptic cortex was used for computing ‘MI z-score’ (grand-median of ‘MI z-score’: 0.96 vs. 0.13; z = 4.30 and p < 0.001 on Wilcoxon signed rank test; Fig. 3A). Sixty-four out of the 76 patients revealed SOZ during extraoperative ECoG recording. In these 64 patients, likewise, SOZ had a greater ‘MI z-score’ compared to non-SOZ (grand-median of ‘MI z-score’: 1.28 vs. 0.52; z = 4.79 and p < 0.001 on Wilcoxon signed rank test; Fig. 3B).

Ninety patients (73%) achieved ILAE Class 1 outcome (mean follow-up period: 5.7 years). The full model provided accurate classification of surgical success (R^2^ = 0.44; p < 0.00001). ‘Incomplete SOZ resection (odds ratio [OR]: 0.03; p = 0.0001)’, and ‘larger number of AEDs (OR: 0.39; p = 0.008)’ decreased the chance of surgical success, whereas ‘larger subtraction MI z-score’ increased it (OR: 1.45; p = 0.01) (Table 1). In other words, each relative increase of ‘MI z-score’ in the resected tissue compared to the preserved tissue increased the odds of surgical success by 45%. The accuracy of outcome classification rated by ROC analysis was AUC of 0.85 (p < 0.00001). When the sensitivity was set to 0.86, the specificity was 0.76 (Fig. 4A).

The model excluding MI also provided an accurate classification of surgical success (R^2^ = 0.27; p = 0.006). ‘Incomplete SOZ resection (odds ratio [OR]: 0.10; p = 0.0005)’, and ‘larger number of AEDs (OR: 0.47; p = 0.01)’ decreased the chance of surgical success (Table 1). The AUC of the ROC plot was 0.77 (p < 0.00001). When the sensitivity was set to 0.86, the specificity was only 0.48 (Fig. 4A).

The model excluding SOZ likewise provided accurate classification of surgical success (R^2^ = 0.23; p = 0.01). ‘Larger number of AEDs (OR: 0.49; p = 0.02)’ and ‘larger size of resection (OR: 0.97; p = 0.05)’ was associated with a reduction of the chance of surgical success, whereas ‘larger *subtraction MI z-score*’ increased the chance (OR: 1.23; p = 0.05). The AUC of the ROC plot was 0.78 (p < 0.00001). When the sensitivity was set to 0.86, the specificity was 0.45 (Fig. 4A).

### Effects of different definitions of the non-epileptic mean on the outcome classification

The accuracy of the outcome classification barely differed among the full models utilizing the non-epileptic mean based on 10, 20, 30, 40, 50, and 60 closest electrode sites. AUC of a given full-model ROC curve remained to be 0.85 regardless of the number of non-epileptic channels to be used for computation of the non-epileptic mean.

### Cross validation of the full model

Employment of a leave-one-out analysis cross-validated the full model’s outcome prediction; namely, the AUC of the ROC plot was 0.77 (p < 0.00001). When the sensitivity was set to 0.86, the specificity was 0.64 (Fig. 4B). Model-based probabilities of surgical success before and after the leave-one-out-method were highly correlated (Pearson correlation coefficient r: 0.98; p < 0.00001).

The result of additional leave-one-out analysis suggested that the model excluding MI had an AUC of 0.61 (p = 0.05) and sensitivity/specificity of 0.86/0.36 (Fig. 4B). Model-based probabilities of surgical success before and after the leave-one-out-method were highly correlated (r: 0.97; p < 0.00001).

Likewise, the model excluding SOZ had an AUC of 0.66 (p = 0.006) and sensitivity/specificity of 0.86/0.27 (Fig. 4B). Model-based probabilities of surgical success before and after the leave-one-out-method were highly correlated (r: 0.97; p < 0.00001).

### Agreement between the model-based probability and the observed frequency of surgical success at an individual patient level

Figure 5A shows a generous agreement between the model-based likelihood of surgical success for given individual patients based on the full model and the observed frequency of surgical success. For example, the full model anticipated that 90 patients would achieve surgical success with a probability of greater than 0.7. Thereby, 80 out of these 90 patients indeed achieved such surgical success. The full model likewise anticipated that 14 patients would achieve surgical success with a probability of smaller than 0.3. Thereby, only two out of these 14 patients indeed achieved surgical success. The full model indicated that the probability of surgical success ranged between 0.3 and 0.7 in the remaining 19 patients; in other words, the full model was unable to classify the surgical outcome with firmness in these 19 patients. Conversely, based on the model excluding MI and that excluding SOZ, the probability of surgical success ranged between 0.3 and 0.7 in 26 and 34 patients, respectively (Fig. 5B,C).

## Discussion

### Methodological innovations and limitations

The current study of 123 patients suggests that precise measurement of the statistical deviation of MI from the anatomically-corresponding non-epileptic mean is technically feasible on invasive recording. Our methodological concept is similar to that of statistical parametric mapping (SPM) most commonly employed in noninvasive neuroimaging and EEG studies^26–28^. The current method allows us to compute ‘MI z-score’ without conducting a region-of-interest (ROI) based analysis as previously reported by our group^24^. Thus, the spatial resolution of the current method for defining the non-epileptic mean was independent of the sizes of pre-set ROIs but of the density of electrode sampling at a given region (Fig. 1A). The sensitivity/specificity of the full regression model including ‘MI z-score’ to classify the seizure outcome was 0.86/0.76, which was comparable to that of the previously-reported model including MI adjusted based on the pre-set ROIs^24,31^. Compared to the raw MI value, the z-score might be easier to signify in the application of MI in presurgical evaluation. Thus, we believe that the current z-score based technique is at least as effective as our previously-reported ROI-based analytic method^24^.

Since extraoperative ECoG recording is tenable only in patients with a focal disease process, regions not involved in the SOZ, interictal spike discharges, or epileptogenic lesions were treated as non-epileptic cortex^44^. Previous studies have demonstrated that non-epileptic cortex of patients with focal epilepsy and that of healthy non-human primates share similar spatial, temporal, and spectral features on task-related ECoG signals^45,46^. Since ECoG recording inevitably suffers from spatial limitations, we computed the non-epileptic mean and standard deviation of MI across 30 closest non-epileptic electrode sites at the FreeSurfer average brain. Selection of 30 such sites for computation of the non-epileptic mean may be appropriate, since the selection of 10, 20, 40, 50 or 60 such sites for this purpose barely altered the outcome classification by the full model. Relatively increased MI in the non-epileptic occipital lobes (Fig. 1B) may be attributed to the abundant occurrence of physiological HFA in these regions during slow-wave sleep^19,20,47^.

The present study did not include children younger than 4 years, since the application of FreeSurfer average brain in such young children has not been validated. In addition, we cannot rule out the possibility that the normative MI value at given anatomical structures might differ between those younger than 4 years and older. We previously demonstrated that there were no significant age-related changes in MI between 4 and 44 years^24^. Conversely, we previously found that MI differ across sleep stages. Specifically, non-REM sleep is associated with modest but significantly increased MI, compared to the other stages^24^. Thus, our normative MI data provided in this study may be better suited to study interictal MI during non-REM sleep. We still do not know if MI during wakefulness is likewise useful for classification of SOZ or postoperative seizure outcome. Our statistical technique was not applicable to major brain malformations such as large porencephaly or hemimegalencephaly, that have been excluded from this study. For such patients with major brain malformations, adjustment of MI might need to be done perhaps using a pre-set ROI^24^.

Successful demonstration of the independent effect of ‘subtraction MI z-score’ in accurate classification of seizure outcome may be attributed to several factors. First of all, this is a single institution study of a large number of patients, all of whom were operated by the same surgeon (S.S). Our logistic regression models did not have to incorporate the effects of ‘institutions’, which might need to be taken into account in multicenter studies. Thus, the present study benefited from a sufficient statistical power to incorporate up to 11 predictor variables. In our previous studies of ECoG recorded with a sampling rate of 200 Hz, cortical regions showing frequent *spike* discharges often turned out to be a part of the SOZ^2^, but ‘complete resection of SOZ’ was the sole independent predictor of postoperative seizure outcome^36^. The occurrence rate of epileptiform discharges is a discrete variable that may be zero in substantial proportions of recording sites in each patient. Thus, measurement of statistical deviation of the occurrence rate of epileptiform discharges from the non-epileptic mean might be more complicated than that of a continuous variable like MI.

The accuracy of outcome classification of the full model was 0.85 as rated by AUC (Figs. 4A and 5A). After a leave-one-out cross-validation process, however, the accuracy of outcome prediction of each new patient dropped to 0.77 (Figs. 4B and 6A). This observation infers that a combination of predictors capable of accurately classifying the outcome within a study cohort of patients may not predict the outcome of a new cohort of patients with similar accuracy. Moreover, the present ECoG study is a retrospective study and by no means suggests that statistical deviation of MI can better localize the epileptogenic zone compared to other interictal spike or HFA measures. It remains to be determined whether the amplitude of HFA, the degree of consistency of coupling with the slow-wave phase, or both is more informative in presurgical evaluation. We are willing to share our ECoG dataset with investigators who wish to test the performance of different HFA analytic methods in predicting SOZ and postoperative seizure outcome. Future collaborative prospective studies are expected to refine the model further to predict seizure outcome so that the results will ultimately have a noteworthy impact on clinical practice.

### Clinical significance

The overall observations support our hypothesis that the statistical deviation of MI from the non-epileptic mean would provide a useful interictal epilepsy biomarker to localize the epileptogenic zone. We computed ‘MI z-score’ at each recording site based on the non-epileptic mean of 47 patients; thereby, ‘MI z-score’ was indeed greater at SOZ compared to at non-SOZ sites in the remaining 76 patients. Resection of regions with MI with a greater deviation (increase) from the non-epileptic mean was associated with better postoperative seizure outcome. The effect of ‘MI z-score’ on outcome classification was significant independently from the effect of clinical, SOZ, and neuroimaging variables. Yet, exclusion of SOZ variables from the regression model resulted in a suboptimal outcome classification, with a substantially lower specificity. In summary, this study provides empirical data supporting the conceptual notion that the epileptogenic zone would be optimally localized not by ictal or interictal measures alone but by the combined consideration of both ictal and interictal epileptic abnormalities^48,49^.

## Data Availability

The data from the present study, including clinical information, ECoG, and MRI, as well as MATLAB-based in-house software are available through the principal investigator (E.A. at easano@med.wayne.edu).
